# Identification of *Vicia ervilia* Germplasm Resistant to *Orobanche crenata*

**DOI:** 10.3390/plants9111568

**Published:** 2020-11-13

**Authors:** Clara Isabel González-Verdejo, Mónica Fernández-Aparicio, Eva María Córdoba, Salvador Nadal

**Affiliations:** 1 IFAPA Centro Alameda del Obispo, Área de Genómica y Biotecnología, Apdo. 3092, 14080 Córdoba, Spain; evam.cordoba@juntadeandalucia.es (E.M.C.); salvador.nadal@juntadeandalucia.es (S.N.); 2 Institute for Sustainable Agriculture, Consejo Superior de Investigaciones Científicas (CSIC), 14004 Córdoba, Spain; monica.fernandez@ias.csic.es

**Keywords:** bitter vetch, parasitic weeds, broomrape, legumes, breeding for *Orobanche* resistance, breeding for tolerance, escape, low germination induction

## Abstract

Bitter vetch (*Vicia ervilia* L.) is an ancient grain legume used as animal feed in the Mediterranean basin. This legume has a large economical potential because of its high yield under low inputs and good protein content, as well as resistance to cold and drought. Nevertheless, its growth and production area are affected in the presence of the broomrape weed species *Orobanche crenata*. Due to the small bitter vetch size, infection by as few as two or three *O. crenata* per vetch plant can be devastating. There are no efficient methods of selectively controlling *O. crenata* in this crop, for which reason the development of varieties resistant and tolerant to *O. crenata* infection is needed. Phytogenetic resources are valuable reserves for species survival. They represent important genetic variability and allow the possibility of finding characters of interest, such as new resistance sources. A large-scale field screening of a collection of 102 bitter vetch accessions indicated that most bitter vetch accessions were susceptible but allowed us to select 16 accessions with low levels of *O. crenata* infection. Next, we used a combination of field and rhizotron experiments to investigate the resistant response of selected bitter vetch genotypes in detail by studying the performance and resistance mechanisms. These experiments led to the identification of three different mechanisms that block *O. crenata* parasitism. A pre-attachment mechanism of low induction of *O.crenata* germination was identified in two bitter vetch accession Ve.055 and Ve.155. In addition, a post-attachment mechanism of resistance to *O. crenata* penetration was identified inthe accession Ve.125. In addition, the field-resistant accession Ve.123 showed susceptible response in rhizotron, indicating that a late mechanism acting after vascular connection, most probably related with bitter vetch of escape due to fructification precocity was acting against *O. crenata* development.

## 1. Introduction

A major problem in Europe is the lack of protein for feeding animals. Almost 63% of the needed protein must be imported and about 48% of the total protein used is soybean [1]. To this problem one must add the market price instability of this raw material, this being the main limiting factor for livestock farming. To sum up, the availability and price of feedstock, normally soybean, are the main issues which determinate future animal feeding in a global market [2]. For these reasons other alternative crops well-adapted to the prevailing agro-climatic conditions and presenting high protein contents are needed for sustainable feeding.

Bitter vetch (*Vicia ervilia* Willd), originating in the Mediterranean and Middle East, is one of the oldest legume crops [3,4]. Bitter vetch is an annual, self-pollinated crop extensively used in the past to add protein in animal feed, however, today it is considered a neglected/underutilized pulse [5] of increasing interest regarding global food security [6]. This interest is two-fold, not only because it represents a good alternative to soybean as a protein source for animal feed but also for its beneficial role providing sustainability to extensive rainfed agro-systems. Bitter vetch can tolerate shallow and low fertility soils [7], and presents low water requirements, growing favourably in environments with low precipitation, being a semi-desert climate-adapted crop. In addition, this crop tolerates cold temperatures [8]. Yields up to 2 t/ha in low rainfall environments are achievable under low input cultivation systems. One study from Spain reported grain yields higher than 3 t/ha [9].

The clear benefits of bitter vetch cultivation are threatened in Mediterranean basin by its high susceptibility to crenata broomrape (*Orobanche crenata* Forsk.). Broomrapes (*Orobanche* and *Phelipanche* species) are obligated holoparasitic weeds that infect many Apiaceae, Asteraceae, Brassicaceae, Fabaceae or Solanaceae crops, thus representing a threat to food security [10,11]. *O. crenata* represents one of the main constraints for cultivation of most grain and forage legumes in the Mediteranean basin [12,13]. Their tiny seeds are distributed in the soil and maintain their germination inhibited until they detect specific host root signals, mainly strigolactones [14]. Once germinated, a radicle develops, grows towards the host root and develops the haustorium, an infection organ that attaches and invade to the host root to connect with crop vascular system to withdraw nutrients and water [15]. Finally, a tubercle is developed where the host-derived water and nutrients are accumulated and from which a floral scape emerges from the soil and gives rise to flowers with the seeds. *Orobanche* control is difficult to achieve due to its high fertility and persistent seedbank and evolution of parasitic populations overcoming the control methods [16]. The current commercial strategies are based in crop resistance and chemical control. Sources of resistance against parasitic weeds are scarce among crop germplasm collections being more frequent in wild relatives to crop species [17]. Resistance mechanisms against the parasitic infection processes of host-induced germination and haustorium development are the most frequent targets in breeding programs [18,19].

Despite the high susceptibility of bitter vetch to *O. crenata* infection, so far no resistant genotypes have been identified. The aim of this study was to examine a collection of 102 bitter vetch accessions and to identify bitter vetch genotypes that are resistant to *O. crenata* infection using a combination of field and rhizotron experiments. In addition, rhizotron experiments were used to characterize the resistant mechanisms involved. The selected material could be the basis for a new breeding program generating locally adapted alternatives with resistance to *O. crenata*.

## 2. Results

### 2.1. Field Screening

**Figure 1:**
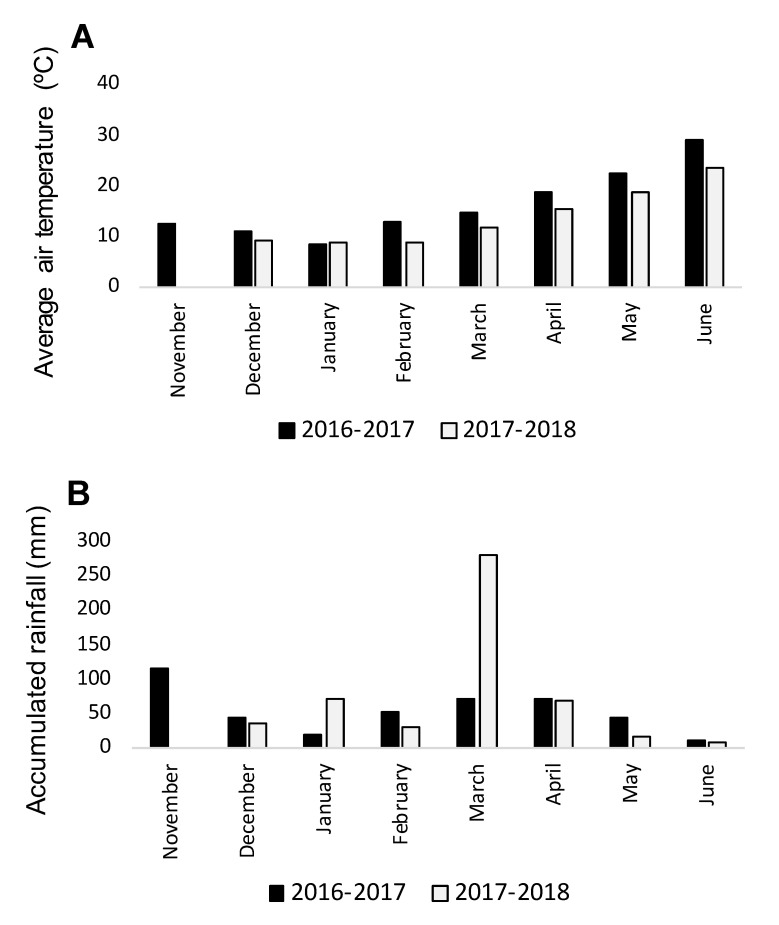
Average air temperature (**A**) and monthly rainfall (**B**) during the growing seasons 2016–2017 and 2017–2018.

In both seasons of field experimentation, weather conditions (Figure 1) were conductive for *O. crenata* infection and its emergence as it is indicated by the high level of *O. crenata* emergence on the faba bean check (average of 2.63 ± 0.14 and of 7.42 ± 0.36 emerged *O. crenata* per faba bean plants during 2016–2017 and 2017–2018 seasons, respectively).

**Figure 2:**
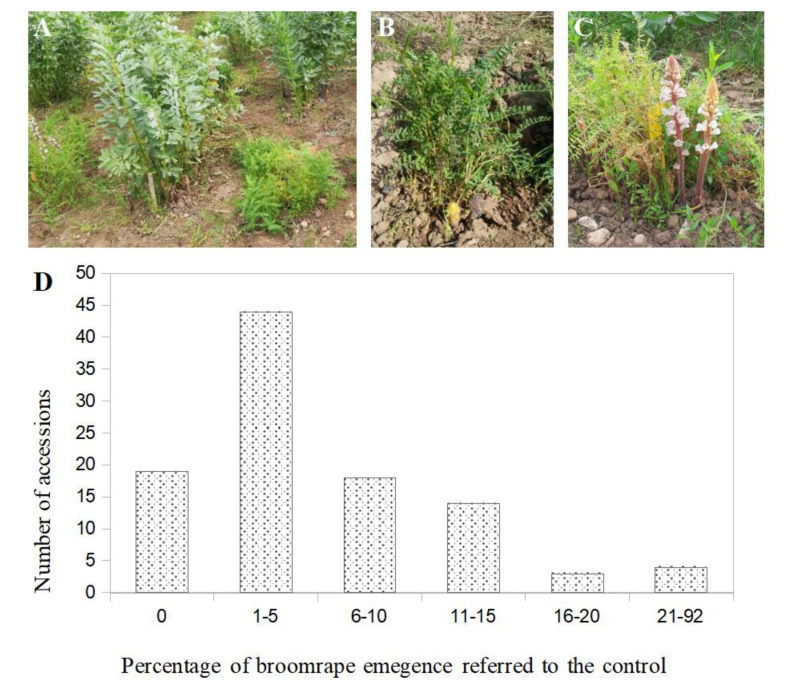
Field experiment carried out to select resistant bitter vetch (*Vicia ervilia*) lines to *Orobanche crenata* (Córdoba, 2016–2017 season): Tested lines were alternately surrounded by the susceptible control faba bean cv Prothabon (**A**). Initial stage of *O. crenata* emergence (**B**) and flowers development (**C**) on bitter vetch plants. Frequency distribution of the 102 bitter vetch accessions studied showing differences in the rate of emergence of broomrape (relative to emergence on the faba bean control) (**D**).

During the first growing season of 2016–2017, bitter vetch lines were in general susceptible to *O. crenata*. The average number of broomrape per bitter vetch plant assessed in the collection of 102 genotypes was 0.12 ± 0.013 ranging from 0.0 in the most resistant accessions to 0.80 ± 0.44 in the most susceptible accessions. In order to avoid errors due to the unavoidable heterogeneous distribution of broomrape seeds across the field trial, the number of emerged broomrape per bitter vetch plant within each row was standardised to the faba bean check. The average ‘broomrape emergence in bitter vetch referred to the faba bean check’ was observed as 6.93 ± 0.01%, ranging from 0.0% in the most resistant accessions to 91.71 ± 0.79% in the most susceptible accessions. The most frequent values were observed among 1 to 5% of infection. Only 19 lines showed no infection by the parasite. Among them, 16 bitter vetch accessions showed a mean value of zero or nearly zero broomrapes per plant while that number in the surrounding faba bean check was high, indicating that the lack of infection was not due to absence of *O. crenata* seeds in the particular area of land that these bitter vetch accessions were growing. The rest of the accessions were infected by the parasite in a range between 1 and 91.71% referred to control (Figure 2).

During the second growing season of years 2017–2018 the selected 16 accessions with the lowest values of infection in 2016–2017 were again studied under field conditions. The Jabegote variety was also included in this assay because of its well known yield data. The average number of broomrape in the 16 bitter vetch plants was 0.72 ± 0.06 ranging from 0.24 ± 0.11 to 1.20 ± 0.11. The average ‘broomrape emergence in bitter vetch referred to the faba bean check’ was observed as 10.17 ± 0.09%, ranging from 3.79 ± 1.57% in the most resistant accessions to 19.20 ± 0.04% in the most susceptible accessions. Ve.55 was the accession with the lower number of emerged *O. crenata* (3.79%) followed by a group of 10 lines with an intermediate rate up to 9.86% of infection. Finally, six lines were the most susceptible showing percentages ranking from 13.39 to 19.20% (Table 1). We found that although line Ve.125 showed in general intermediate susceptibility to *O. crenata*, a unique plant belonging to this accession resulted to be tolerant to the parasite. For this reason it was selected for deeper studies in rhizotron. No relationship between the origin of the lines and its susceptibility to *O. crenata* infection was found.

In addition to the number of emerged broomrape at host maturity, additional parameters of broomrape infection were assessed in the second field growing season. The cumulative broomrape dry matter per bitter vetch plants averaged as 1.27 ± 0.10 g/bitter vetch plant and the average weight of individual parasites in bitter vetch averaged 0.14 ± 0.01 g/broomrape plant. The weight of individual parasites was not statistically significant among lines (*p* < 0.05) indicating an homogeneous parasite size. The relative parasite weight (ratio between parasite biomass and combined bitter vetch-parasite biomass) averaged 0.35% ± 0.03. For those parameters of infection there were significant differences among bitter vetch accessions. The total parasitic biomass per bitter vetch plant averaged from 0.63 ± 0.30 g/bitter vetch plant in accession Ve.68 to 1.99 ± 1.10 g/bitter vetch plant in accession Ve.153. The weight of a single parasite growing per plant in each bitter vetch accession averaged from 0.06 ± 0.01 g/broomrape plant in accession Ve.077 to 0.22 ± 0.11 g/broomrape plant in accession Ve.080. The relative parasitic weight (ratio between parasite biomass and combined bitter vetch-parasite biomass) averaged from 0.13% ± 0.06 in accession Ve.055 to 0.50% ± 0.23 in accession Ve.153.

**Figure 3:**
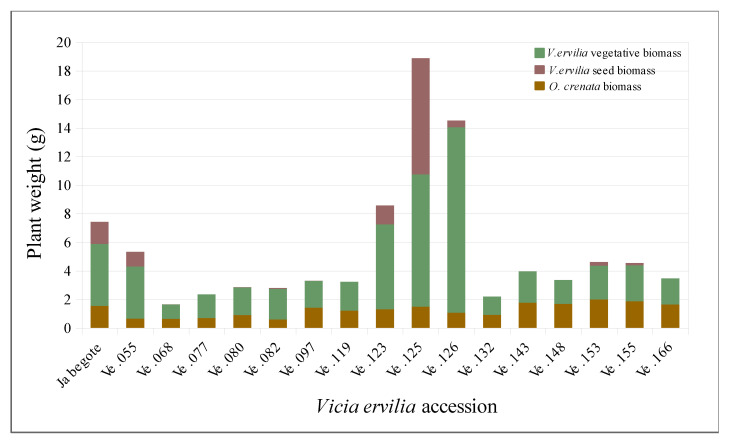
Dry matter in the pathosystem *Vicia ervilia*-*Orobanche crenata*. Host dry matter (detailed as vegetative and reproductive host weight), as well as cumulative parasite dry matter are given for the selected bitter vetch lines during 2017–2018 season.

Bitter vetch productivity was also characterized as total host dry matter (vegetative and reproductive host aboveground tissues) which averaged 4.26 ± 1.18 and host reproductive index (ratio between host reproductive dry matter and total aboveground host dry matter) which averaged 0.05 ± 0.01. Total host dry matter averaged from 1.02 ± 0.67 in line Ve.068 and 17.38 ± 15.42 in line Ve.125 (Figure 3). Host reproductive index averaged from 0 in lines Ve.119, Ve.132, Ve.143, Ve.148 and Ve.166 to 0.21±0.01 in Ve.055. To identify mechanisms of escape in bitter vetch to *O. crenata* infection the relationship between bitter vetch precocity and level of infection was studied in the germplasm collection. Data on GDD to flowering from each bitter vetch accession was regressed with (i) broomrape emergence per bitter vetch plant, (ii) cumulative broomrape dry matter per bitter vetch plant, and (iii) the relative parasitic weight. GDD to flowering in the bitter vetch lines averaged 1073.70 ± 22.49 °C, ranging from 914.60 °C for both lines Ve.055 and Ve.153 to 1321.40 °C for line Ve.080. Flowering in lines Ve.077 and Ve.148 was not observed (Table 2).

The results showed a high correlation among bitter vetch GDD to flowering and the three *O. crenata* parameters considered: broomrape emergence per bitter vetch plant (0.87), cumulative broomrape dry matter per bitter vetch plant (0.88) and the relative parasitic weight (0.87) (*p* < 0.05), indicating a direct relationship between host precocity and low parasitic infection.

### 2.2. Rhizotron Screening

**Figure 4:**
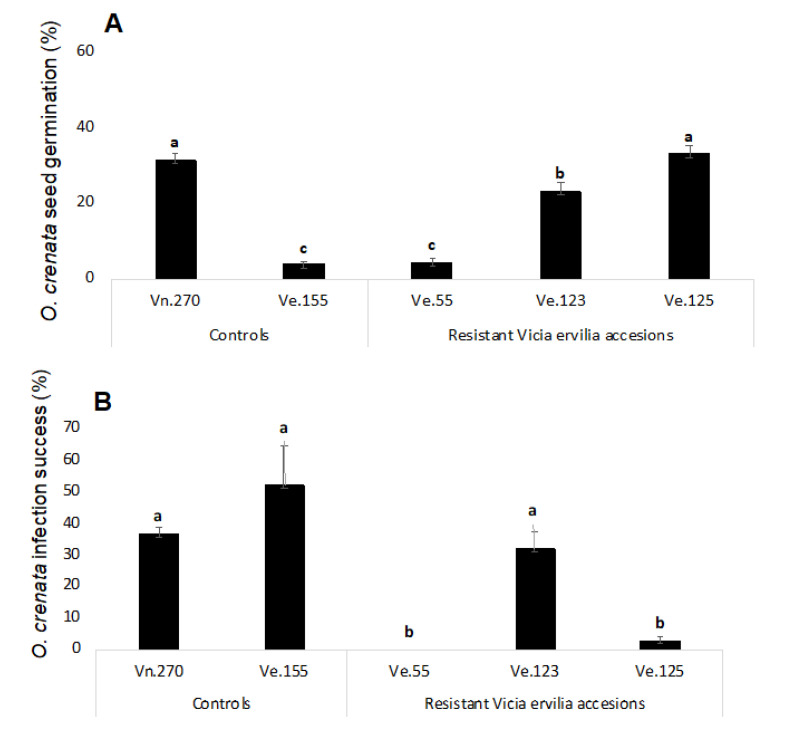
Resistant response of selected bitter vetch accessions Ve.55, Ve.123, Ve.125 in rhizotron experiments. (**A**) Induction of *Orobanche crenata* germination; (**B**) Infection success of *Orobanche crenata* radicles. The same letter in each figure indicates that differences are not statistically significant (LSD test, *p* < 0.05). Error bars represent the mean + s.e.

A rhizotron experiment was used to characterize the mechanisms involved in the resistant response to *O. crenata* infection observed in field in the accessions Ve.055, Ve.123 and Ve.125. The rhizotron experiment used two susceptible controls, the susceptible bitter vetch accession Ve.155 and susceptible control *Vicia narbonensis* Vn.270 [20]. *O. crenata* seeds located at a distance of 3 mm from the vetch roots were inspected under a stereoscopic microscope to determine the percent of *O. crenata* germination (Figure 4A). The higher levels of *O. crenata* germination were observed in the narbon bean control and in the resistant bitter vetch accessions Ve.123 and Ve.125. Interestingly, roots of the accession Ve.055 induced very low levels of *O. crenata* germination which indicates that a resistant mechanism is acting in this bitter vetch accession either through low exudation of *O. crenata* germination stimulants or exudation of *O. crenata* germination inhibitors at *O. crenata* pre-attachment stage. Low levels of *O. crenata* germination was also found in the susceptible control Ve.155 reason by which other susceptibility mechanism is expected to be found in the following stages of the parasitic plant development.

The infection success of *O. crenata* radicles to infect each vetch genotype was assessed by observing under a stereoscopic microscope the germinated *O. crenata* seeds located at a distance of 3 mm from the bitter vetch roots and determining the percent of *O. crenata* radicles in contact to vetch roots that successfully penetrated the roots and formed a healthy tubercle (Figure 4B). A high level of infection success was observed in the susceptible control Vn.270 and in the resistant accession Ve.123 (36.70 ± 2.09 and 32.00 ± 5.65 respectively). *O. crenata* seeds stimulated by Ve.123 line not only were able to germinate, but also to successfully infect the host plant. Since this line presented a precocious flowering (GDD 1170.4 °C), a mechanism of escape could be acting allowing the plant to trigger the development of the seeds before parasite establishment.

**Figure 5:**
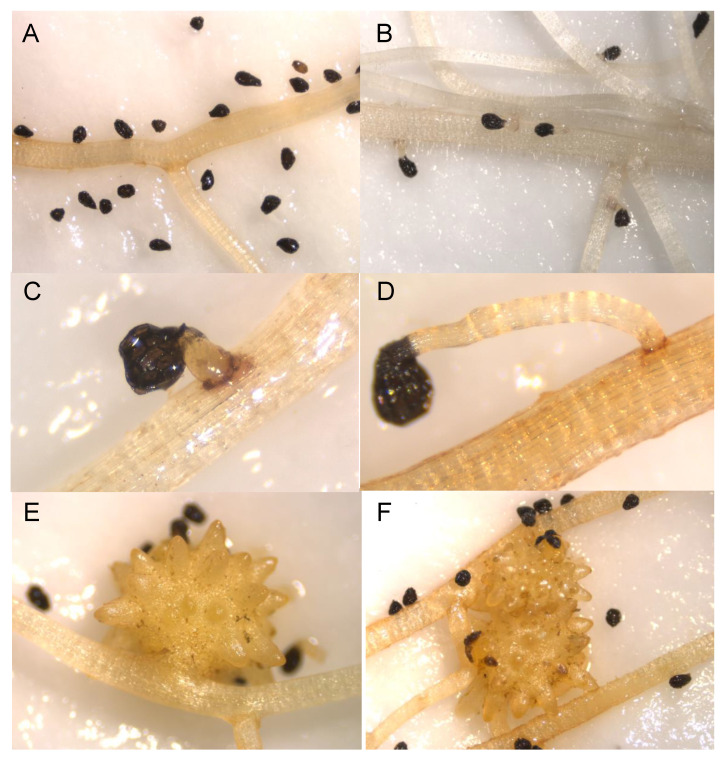
Differences during the infection process between resistant and susceptible bitter vetch genotypes: (**A**) Resistance based in low broomrape germination induction of Ve.55 roots. (**B**–**D**) Resistance based in inhibition of broomrape penetration of Ve.125 roots. (**E**) Induction of broomrape germination by roots of Ve.123, without resistance to penetration. (**F**) Low induction of broomrape germination by roots of Ve.155 with high susceptibility to penetration of germinated broomrape seedlings.

Despite the low induction activity of *O. crenata* germination observed in the susceptible bitter vetch accession Ve.155, the few *O. crenata* seeds that germinated and contacted this Ve.155 accession were able to infect with higher success than those *O. crenata* radicles that contacted the roots of the narbon bean susceptible controls. This fact could possibly indicate that exudation of allelochemicals inhibiting the *O. crenata* development may not be responsible for the low germination activity observed in this Ve.155 accession. The few *O. crenata* seeds that germinated with the roots of the second bitter vetch accession with low germination activity (Figure 5). Ve.055, were not able to contact the root and therefore, no penetration and tubercle formation was observed in this bitter vetch accession. The third bitter vetch accession with resistant response in field, the accession Ve.125 which induced in rhizotron high levels of germination in *O. crenata* seeds, allowed however low levels of infection. The emerged radicles from the *O. crenata* seeds stimulated by roots of Ve.125 were not able to infect, being observed in this bitter vetch accession very low levels of infection success (2.86 ± 1.26) indicating the existence of second resistant mechanism in the bitter vetch species, acting at post-attachment level during the bitter vetch root penetration process of *O. crenata*. *O. crenata* tubercles formed in all vetch accessions, were inspected to detect necrosis. Tubercle necrosis was not observed in any of the vetch lines studied in this work.

## 3. Discussion

*Orobanche* management based in breeding for *Orobanche* resistance is a straightforward strategy when good sources of vertical and monogenic resistance are available among germplasm collections of crop species [21]. Unfortunately, this is not true for most of the crop species infected by *Orobanche* weeds, except for the interaction of sunflower-*O. cumana* were genetic resistance has been found to be vertical, following a gene-for-gene interaction where dominant resistant genes are easily incorporated into agronomically-interesting cultivars [22]. Usually, only incomplete resistance levels generally characterised by a reduced number of emerged broomrapes has been identified in other crops such as tomato, oilseed rape, and legumes. In these crops, resistance against broomrapes is usually scarce and of complex quantitative nature [12]. Among the scarce resistance identified so far in various crops, a range of mechanisms of resistance have been identified, each blocking subsequent infection stages [23,24] at root surface pre-attached and post-attached haustorial stages of the infection cycle [17,25]. Pyramiding genes each blocking successive stages in the infection cycle will increase the durability of resistant cultivars against the evolution of new populations of broomrape with enhanced virulence [16].

Despite the high susceptibility of bitter vetch to *O. crenata* infection, no resistant genotypes have been identified so far. In this study, the use of a combination of field and rhizotron experiments, have allowed us to identify and characterize for the first time two resistant and one tolerant bitter vetch genotypes among a large germplasm collection. The identified resistant phenotypes were similar in field but different when the interaction of their roots with *O. crenata* seeds and seedlings were studied under stereoscopic microscope in rhizotron, highlighting the importance of combining field and rhizotron screening protocols in breeding for *Orobanche* resistance [21,26].

In this work we report in bitter vetch a pre-attachment resistance mechanism of low induction of *O. crenata* germination in two bitter vetch genotypes Ve.055 and Ve.155. The low induction of *O. crenata* germination translated into low level of root infection in rhizotron and field in the bitter vetch genotype Ve.055 but not in the Ve.155 due to increased susceptibility of Ve.155 roots to infection by the few seeds that germinated, highlighting the susceptibility of this resistance mechanism to be overcome by virulence-enhanced *Orobanche* populations, and the importance of pyramiding this mechanism with others acting at later stages of the infection process during the root penetration stages. Low germination-inducers genotypes have been described previously in faba been [21,27,28], tomato [29,30] and sunflower [24,31]. This resistance mechanism has been revealed useful in crop breeding against parasitic weeds [19,32].

In this work we also report in bitter vetch a second resistance mechanism of post-attachment resistance in the accession Ve.125 acting during the bitter vetch root penetration process of *O. crenata* haustorium. Resistance to radicle infection has been described previously in faba bean [33]. Resistant genotypes can block the parasite intrusion process at either the root cortex, endodermis or central cylinder by creating physical barriers such as lignification, suberization of protein cross-linking that block the growth of the haustorium inside the host root and chemical barriers such as excretion of phytoalexins that poison the parasite inside the host root [34]. Although histological studies were out of the scope of the present manuscript, the histological characterization of the Ve.125 post-attachment mechanisms that blocks the intrusion of the *O. crenata* haustorium through the Ve.125 root and inhibits formation of *O. crenata* tubercle will be performed in the near future.

Despite the low level of emerged *O. crenata* on Ve.123 during both years of field experiments, rhizotron experiments revealed that roots of this bitter vetch genotype are susceptible to *O. crenata* infection, and no mechanisms of pre-attachment or post-attachment are acting. *O. crenata* tubercles formed on roots of Ve.123 were healthy and no necrosis was observed on them until the end of the rhizotron experiment indicating that Ve.123 could be tolerating the *O. crenata* infection by a mechanism that makes the host sinks more competitive that the parasitic sink strength. We found that Ve.123 genotype set the reproductive stage early and Ve.123 pot filling could be competing for photoassimilates reallocation with the parasitic sink, better than other late bitter vetch genotypes. A mechanism of escape due to early flowering play a major role reducing the *Orobanche* infection after tubercle development [35,36].

The different resistant mechanisms identified in this work for the first time in *Vicia ervilia* species act at successive stages during the infection process of *O. crenata* and could be accumulated in a single variety by crossing the accessions Ve.055 with Ve.125 to obtain a gene-pyramid bitter vetch line with more durable resistant against *O. crenata* [16]. Crossing the accessions Ve.123 and Ve.125 would also be interesting since it could result into a resistant line with high productivity. The use of molecular markers linked to specific resistance mechanisms will allow tracking them in segregating populations facilitating their pyramiding with other genes [37].

## 4. Material and Methods

### 4.1. Plant Material

A set of 102 accessions of a *V. ervilia* germplasm collection was studied in order to identify resistance to *O. crenata*. This material was kindly provided by Centro de Recursos Fitogenéticos (Madrid, Spain), the Genetic Resources Unit of U. S Department of Agriculture (Pullman, WA, USA) and the IFAPA legume germplasm bank (BGLI, Córdoba, Spain). For rhizotron experiments, broomrape seeds were collected from dry inflorescences of *Orobanche crenata* plants growing on *Vicia sativa* using a 0.6 mm mesh-size sieve (Filtra, Barcelona, Spain) and stored dry in the dark at 4 °C until use.

### 4.2. Field Experiments

A total of 102 bitter vetch accessions were evaluated for a first screening in a field naturally infested with *O. crenata* seeds at the IFAPA Alameda del Obispo Center (Cordoba, Spain). The experiments were carried out during 2016–2017 season, being the sowing date the 11st November 2016. Each bitter vetch accession was sown in a row of 0.5 m with 25 seeds per row and a distance between rows of 1 m with three replicates in a randomized complete block design. Since there is no information about bitter vetch-susceptible lines, each bitter vetch row was alternately surrounded by rows of the susceptible control faba bean cv Prothabon [38]. Broomrape infection was assessed at crop maturity counting the number of emerged broomrapes per plant within each row. In order to avoid the error due to the unavoidable heterogeneous distribution of broomrape seeds across the trial, the number of emerged broomrape per host plant within each row was standardised as ‘broomrape emergence referred to the checks as percentage of the mean value of the four surrounding rows of faba bean control [35]. Bitter vetch lines were only considered when they showed a mean value of zero or nearly zero broomrapes per plant but that number was high in the control.

### 4.3. Field Experiments under High Infestation Conditions

For a more detailed study of the resistance response observed in the first field trial, the 16 accessions showing the lowest values of ‘broomrape emergence referred to the check’ across the first field experiment were studied in a second field experiment performed during the growing season 2017–2018 at the IFAPA Alameda del Obispo Center. The sowing date was the 22nd December 2017 and, as in the previous experiment each line was sown in a row of 0.5 m with 25 seeds per row in three repetitions. Bitter vetch accessions were cultivated under natural infestation conditions but in a specific place in the ground with higher and very uniform broomrape seed infestation. Since the soil area is limited in this case, the number of accessions that can be evaluated is far fewer. Nevertheless, this approach allows a more accurate evaluation of the selected lines avoiding the error due to the inevitable heterogeneous distribution of broomrape seeds that usually occurs in broomrape field experiments. Bitter vetch cv Jabegote was also included in the study due to its previously characterized yield data. The susceptible control faba bean cv Prothabon was included as a control as described above in order to normalize the broomrape infection data for each bitter vetch test row. The number of emerged broomrape per each bitter vetch row was standardised as ‘broomrape emergence referred to the check’ as percentage of the mean value of the four surrounding rows of faba bean control [35].

In addition to final number of emerged broomrape, the host-*O. crenata* interaction was studied in the most contrasting lines by using additional parameters as described by Fernández-Aparicio et al. [13]: (i) cumulative parasitic dry matter per host plant (total dry biomass of emerged broomrapes per host plant) (ii) average weight of individual parasites in each host plant (cumulative broomrape dry matter /number of broomrapes per host plant) (iii) total host dry matter (vegetative and reproductive bitter vetch aboveground tissues) (iv) combined biomass (host and parasite biomass) (v) relative parasitic weight (ratio between parasite biomass and combined biomass), and (vi) host reproductive index (ratio between host reproductive dry matter and total aboveground host dry matter).

### 4.4. Escape to O. crenata Infection Observed in the Bitter Vetch Germplasm Collection

To identify mechanisms of escape in bitter vetch to *O. crenata* infection the relationship between bitter vetch precocity and level of infection was studied in the germplasm collection. Air temperature collected at the weather station at IFAPA Alameda del Obispo Center [39] was converted to cumulative growing degree-days (GDD) for *n* days to flowering of each bitter vetch accession according to McMaster and Wilhelm [40]. GDD to flowering from each bitter vetch accession was regressed with (i) number of broomrape emerged per plant, (ii) cumulative broomrape dry matter per bitter vetch plants, and (iii) the relative parasitic weight.

### 4.5. Rhizotron Assay 

In order to validate the results obtained in field and characterize the resistance mechanisms involved, the interaction of roots of bitter vetch with *O. crenata* was studied using rhizotron experiments [41]. The *O. crenata* infection on roots of bitter vetch accessions presenting both values of high resistance in field as well as high reproductivity ability even in infected conditions (Ve.55, Ve.123 and Ve.125) were compared to the *O. crenata* infection of roots of the susceptible control *Vicia narbonensis* Vn.270 [20] as well as one bitter vetch accession with susceptible response in the field (Ve.155).

Bitter vetch seeds were surface sterilized with 2% (*w/v*) NaOCl solution and 0.02% (*v/v*) Tween 20 for 5 min and then rinsed thoroughly with sterile distilled water and germinated in 9 cm diameter Petri dishes with moistened filter papers placed for 4 days in a growth chamber under dark warm (23 °C) conditions before the setting of each experiment. Five bitter vetch seedlings were individually transferred to glass fiber filter paper (GFFP) sheets (Whatman International Ltd., Maidstone, UK) and placed over square Petri dishes (12 cm by 12 cm) filled with sterile perlite moistened with sterile distilled water. Petri dishes were previously punctured on the top to allow bitter vetch stems to develop outside the dish. Seeds of *O. crenata* were surface sterilized by immersion in 0.5% (*w/v*) NaOCl and 0.02% (*v/v*) Tween 20, for 5 min, rinsed thoroughly with sterile distilled water, spread separately over GFFP sheets (12 sheets for each population) at a density of 50 seeds cm^2^ and stored in the dark for 10 days to allow *O. crenata* seed conditioning. Then, the GFFP sheets containing the roots of each bitter vetch accession were replaced by GFFP sheets containing the conditioned *O. crenata* seeds allowing simultaneous reception of germination stimulants in the *O. crenata* seeds. The Petri dishes containing bitter vetch-*O. crenata* cocultivation system were sealed with Parafilm, wrapped in aluminum foil and stored vertically in a growth chamber (23/20 °C, 16/8 h day/night). Plants received Hoagland’s nutrient solution [42] modified at one-quarter strength once per week. *O. crenata* seeds located at a distance of 3 mm from the bitter vetch roots were inspected under a stereoscopic microscope to determine: (i) the percent of *O. crenata* germination; (ii) the percent of contacted *O. crenata* radicles that successfully penetrated bitter vetch root and formed a healthy tubercle and (iii) percent of total formed tubercles that died at early stages of development.

### 4.6. Statistical Analysis

The experimental design was randomized complete blocks. The data were analysed by analysis of variance (one-way ANOVA) with accession as the main factor using Statistix 9.1 software (Analytical software, Tallahassee, FL, USA). The significance of mean differences among accessions was evaluated by the least significant difference (LSD) (*p* < 0.05). Percentages of broomrape germination data were transformed with arcsin (√(x/100) before analysis.

## 5. Conclusions

The majority of bitter vetch accessions tested in our general field screening showed susceptible responses to *O. crenata* infection, confirming the high susceptibility of this species to this root parasitic weed. A second field experiment with a selected set of bitter vetch accessions grown under dense and uniform *O. crenata* seed bank allowed us to characterize the severe biomass loss produced by *O. crenata* infection in susceptible accessions, a negative correlation between precocity and number of emerged *O. crenata* and to detect four bitter vetch accessions that exhibited a high level of field resistance, with no emerged broomrapes and did not suffer from biomass low. Further rhizotron studies allowed the dissection of resistance components acting at successive steps during the *O. crenata* infection. To the best of our knowledge this is the first report describing resistance of bitter vetch genotypes against *O. crenata*.

## Figures and Tables

**Table 1:** Emerged broomrape in field as percent of faba bean control on selected bitter vetch lines and the commercial bitter vetch variety Jabegote during 2017–2018 season. The weight of single parasite growing per plant is detailed.

Accession	USDA Code	Origin	% Emerged Broomrape in Field	Weight of Individual Parasite (g)
Ve.166	PI632671	Turkey	19.20 ± 4.10 a	0.13 ± 0.01
Ve.143	PI518465	Spain	15.43 ± 2.96 ab	0.09 ± 0.02
Jabegote			14.43 ± 6.23 abc	0.15 ± 0.03
Ve.155	PI628293	Bulgaria	13.79 ± 2.03 abcd	0.10 ± 00
Ve.097	PI268476	Afghanistan	13.41 ± 2.05 abcd	0.10 ± 0.02
Ve.077	PI223417	Iran	13.39 ± 10.29 abcde	0.06 ± 0.00
Ve.153	PI628291	Bulgaria	9.86 ± 3.03 bcde	0.16 ± 0.03
Ve.148	PI577716	Bulgaria	9.73 ± 2.93 bcde	0.11 ± 0.00
Ve.125	PI422497	Germany	9.46 ± 2.08 bcde	0.14 ± 0.01
Ve.082	PI228296	Iran	8.26 ± 4.75 bcde	0.13 ± 0.03
Ve.080	PI227471	Iran	7.98 ± 1.68 bcde	0.22 ± 0.11
Ve.132	PI515983	Turkey	7.78 ± 1.02 bcde	0.13 ± 0.01
Ve.068	PI222213	Afghanistan	7.77 ± 3.73 bcde	0.08 ± 0.03
Ve.119	PI393850	Canada	7.19 ± 1.70 cde	0.13 ± 0.01
Ve.126	PI426201	Afghanistan	6.83 ± 1.37 cde	0.16 ± 0.00
Ve.123	PI393854	Canada	5.74 ± 0.77 de	0.22 ± 0.02
Ve.055	PI203145	Jordan	3.79 ± 0.16 e	0.22 ± 0.08

The same letter per column indicates that differences are not statistically significant (LSD test, *p* < 0.05). ± (Standard error mean).

**Table 2:** Growing degree-days (GDD) to flowing on selected bitter vetch lines and the commercial bitter vetch variety Jabegote during 2017–2018 season.

Accession	GDD
Jabegote	945.90 ± 31.30
Ve.055	914.60 ± 0
Ve.068	1008.50 ± 0
Ve.077	n.o.
Ve.080	1321.40 ± 0
Ve.082	1008.50 ± 0
Ve.097	1170.40 ± 0
Ve.119	1170.40 ± 0
Ve.123	1170.40 ± 0
Ve.125	1008.50 ± 0
Ve.126	1008.50 ± 0
Ve.132	1321.40 ± 0
Ve.143	1170.40 ± 0
Ve.148	n.o.
Ve.153	914.60 ± 0
Ve.155	1031.20 ± 74.71
Ve.166	1245.90 ± 75.50

± (Standard error mean).n.o. (Flowering was not observed)

## References

[1] (2018). COM(2018) 757 Final. Report from the Commission to the Council and the European Parliament on the Development of Plant Proteins in the European Unión.

[2] Farrel D.J. (2005). Matching poultry productions with available feed resources: Issues and constrains. Worlds Poult. Sci. J..

[3] Ladizinsky G. (1998). Plant Evolution under Domestication.

[4] Zohary D., Hopf M. (2000). Domestication of Plants in the Old World.

[5] López-Bellido L., Hernándo Bermejo J.E., León J. (1994). Neglected crops: 1492 from a different perspective. Plant Production and Protection Series No. 26.

[6] Jaenicke H., Hoschle-Zeledon I. (2006). Strategic Framework for Underutilized Plant Species Research and Development, with Special Reference to Asia and the Pacific, and to Sub- Saharan Africa.

[7] López Bellido L., Hernando Bermejo J.E., León J. (1994). Grain Legumes for animal feeds. Neglected Crops: 1492 from Different Perspective.

[8] Abd El Moneim A.M. (1993). Agronomic potential of three vetches (*Vicia* spp.) under Rainfed conditions. J. Agron. Crop Sci..

[9] Enneking D., Francis C.M. (2007). Development of *Vicia ervilia* as a grain crop for Southern Australia. Center for Legumes. Mediterranean Agriculture.

[10] Parker C., Riches C.R. (1993). Parasitic Weeds of the World: Biology and Control.

[11] Parker C. (2009). Observations on the current status of *Orobanche* and *Striga* problems worldwide. Pest Manag. Sci..

[12] Rubiales D., Fernández-Aparicio M. (2012). Innovations in parasitic weeds management in legume crops. Agron. Sustain. Dev..

[13] Fernández-Aparicio M., Flores F., Rubiales D. (2016). The effect of *Orobanche crenata* infection severity in faba bean, field pea and grass pea productivity. Front. Plant Sci..

[14] Xie X., Yoneyama K., Yoneyama K. (2010). The strigolactone story. Annu. Rev. Phytopathol..

[15] Riopel J.L., Timko M.P., Press M.C., Graves J.D. (1995). Haustorial initiation and differentiation. Parasitic Plants.

[16] Fernández-Aparicio M., Delavault P., Timko M. (2020). Management of infection by parasitic weeds: A review. Plants.

[17] Scholes J.D., Press M.C. (2008). Striga infestation of cereal crops–an unsolved problem in resource limited agriculture. Curr. Opin. Plant Biol..

[18] Yoder J.I., Scholes J.D. (2010). Host plant resistance to parasitic weeds; recent progress and bottlenecks. CurrOpin Plant Biol..

[19] Fernández-Aparicio M., Westwood J.H., Rubiales D. (2011). Agronomic, breeding and biotechnological approaches for parasitic plant management by manipulating strigolactone levels in agricultural soils. Bot. Bot..

[20] Nadal S., Cubero J.I., Moreno M.T. (2007). Sources of resistance to broomrape (*Orobanche crenata* Forsk.) in narbon vetch. Plant Breed..

[21] Fernández-Aparicio M., Moral A., Kharrat M., Rubiales D. (2012). Resistance against broomrapes (*Orobanche* and *Phelipanche* spp.) in faba bean (*Viciafaba*) based in low induction of broomrape seed germination. Euphytica.

[22] Fernández-Martínez J.M., Pérez-Vich B., Velasco L., Martínez-Force E., Dunford N.T., Salas J.J. (2015). Sunflower broomrape (*Orobanchecumana* Wallr.). Sunflower Oilseed. Chemistry, Production, Processing and Utilization.

[23] Jorrín J., Pérez-de-Luque A., Serghini K., Cubero J.I., Moreno M.T., Rubiales D., Sillero J.C. (1999). How plants defend themselves against root parastic angiosperms: Molecular studies with Orobanchespp. Resistance to Orobanche: The State of the Art.

[24] Labrousse P., Arnaud M.C., Serieys H., Bervillé A., Thalouan P. (2001). Several mechanisms are involved in resistance of Helianthus to *Orobanche Cumana* Wallr. Ann. Bot..

[25] Timko M., Scholes J. (2013). Host reaction to attack by root parasitic plants. Parasitic Orobanchaceae: Parasitic Mechanisms and Control Strategies.

[26] Fernández-Aparicio M., Sillero J.C., Pérez-de-Luque A., Rubiales D. (2008). Identification of sources of resistance to crenate broomrape (*Orobanche crenata*) in Spanish lentil (*Lens culinaris*) germplasm. Weed Res..

[27] Abbes Z., Kharrat M., Simier P., Chaıbi W. (2007). Characterisation of resistance to crenate broomrape (Orobanchecrenata) in a new small seeded line of Tunisian faba beans. Phytoprotection.

[28] Fernández-Aparicio M., Kisugi T., Xie X., Rubiales D., Yoneyama K. (2014). Low strigolactone root exudation: A novel mechanism of broomrape (*Orobanche* and *Phelipanche* spp.) resistance available for faba bean breeding. J. Agric. Food Chem..

[29] El-Halmouch Y., Benharrat H., Thalouarn P. (2006). Effect of root exudates from different tomato genotypes on broomrape (*O. aegyptiaca*) seed germination and tubercle development. Crop Prot..

[30] Dor E., Alperin B., Wininger S., Ben-Dor B., Somvanshi V.S., Koltai H., Kapulnik Y., Hershenhorn J. (2010). Characterization of a novel tomato mutant resistant to *Orobanche* and *Phelipanche* spp. weedy parasites. Euphytica.

[31] Serghini K., Pérez-de-Luque A., Castejón-Muñoz M., García-Torres L., Jorrín J.V. (2001). Sunflower (*Helianthus annuus* L.) response to broomrape (*Orobanche cernua* Loefl.) parasitism: Induced syn- thesis and excretion of 7-hydroxilated simple coumarins. J. Exp. Bot..

[32] Ejeta G. (2007). Breeding for *Striga* resistance in sorghum: Exploitation of an intricate host-parasite biology. Crop Sci..

[33] Rubiales D., Rojas-Molina M.M., Sillero J.C. (2016). Characterization of Resistance Mechanisms in Faba Bean (*Vicia faba*) against Broomrape Species (*Orobanche* and *Phelipanche* spp.). Front. Plant Sci..

[34] Pérez-de-Luque A., Fondevilla S., Pérez-Vich B., Aly R., Thoiron S., Simier P., Castillejo M.A., Fernández J.M., Jorrín J., Rubiales D. (2009). Understanding *Orobanche* and *Phelipanche*-host plant interactions and developing resistance. Weed Res..

[35] Fernández-Aparicio M., Flores F., Rubiales D. (2009). Field response of *Lathyrus cicera* germplasm to crenate broomrape (*Orobanche crenata*). Field Crops Res..

[36] Fernández-Aparicio M., Flores F., Rubiales D. (2012). Escape and true resistance to crenate broomrape (*Orobanchecrenata* Forsk.) in grass pea (*Lathyrus sativus* L.) germplasm. Field Crops Res..

[37] Fondevilla S., Fernández-Aparicio M., Satovic Z., Emeran A.A., Torres A.M., Moreno M.T., Rubiales D. (2010). Identification of quantitative trait loci for specific mechanisms of resistance to *Orobanche crenata* Forsk. In pea (*Pisum sativum* L.). Mol. Breed..

[38] Rubiales D., Pérez-de-Luque A., Cubero J.I., Sillero J.C. (2003). Crenate broomrape (*Orobanche crenata*) infection in field pea cultivars. Crop Prot..

[39] RIA (Red Agroclimática de Andalucía). https://ifapa.junta-andalucia.es/agriculturaypesca/ifapa/riaweb/web/.

[40] McMaster G.S., Wilhelm W.W. (1997). Growing degree-days: One equation, two interpretations. Agric For. Meteorol..

[41] Fernández-Aparicio M., Sillero J.C., Rubiales D. (2009). Resistance to broomrape species (*Orobanche* spp.) in common vetch (*Vicia sativa* L.). Crop Prot..

[42] Hoagland D.R., Arnon D.I. (1950). The Water-culture Method for Growing Plants without Soil. Californian Agricultural Experimental Station. Circular No. 347.

